# Removal of the intrahepatically migrated and impacted plastic stent over malignant stricture using drill dilator

**DOI:** 10.1055/a-2626-3659

**Published:** 2025-07-10

**Authors:** Daiki Yamashige, Susumu Hijioka, Yoshikuni Nagashio, Shota Harai, Mark Chatto, Yutaka Saito, Takuji Okusaka

**Affiliations:** 1 68380 Department of Hepatobiliary and Pancreatic Oncology, National Cancer Center Hospital, Tokyo, Japan; 2 37571 Department of Medicine, Makati Medical Center, Manila, Philippines; 3 Endoscopy Division, National Cancer Center Hospital, Tokyo, Japan

Endoscopic retrograde cholangiopancreatography-guided stent placement is widely performed; however, stent migration can occur. Migrated stents are typically retrieved using grasping forceps or balloon catheters. However, these conventional devices sometimes fail.

We report a case in which an intrahepatically migrated plastic stent (PS) was successfully retrieved using a drill dilator (Tornus ES, Olympus Co., Japan).

An 84-year-old man with resectable perihilar cholangiocarcinoma (Bismuth type IIIa) developed jaundice. A 7-Fr PS had been previously placed in the left hepatic duct across the papilla. The patient later developed cholangitis. Computed tomography revealed that the PS had migrated toward the hepatic hilum ( Fig. 1 **a** ).

**Fig. 1:**
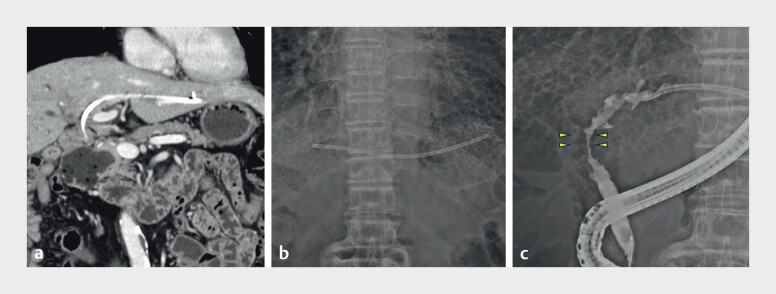
Intrahepatically migrated and stuck plastic stent over the malignant stricture. **a** Computed tomography image showing a plastic stent (straight type, 7 Fr) deployed for resectable malignant hilar biliary obstruction migrating to the left intrahepatic duct over the main stricture. **b** In the fluoroscopic image taken immediately before endoscopic retrograde cholangiopancreatography, the plastic stent has migrated to the deeper side of the left intrahepatic duct. **c** The plastic stent has migrated over the main malignant stricture (yellow arrows) due to hilar cholangiocarcinoma.

Fluoroscopy confirmed that the stent had migrated deeper into the intrahepatic duct beyond the stricture ( Fig. 1 **b, c** ). Attempts to retrieve the stent using a balloon catheter were unsuccessful due to its impaction. Additionally, grasping forceps could not pass through the strictures. Consequently, we opted to use a drill dilator for stent removal ( Video 1 ).

An intrahepatically migrated stent in the intrahepatic duct beyond a malignant stricture, which could not be removed with conventional devices, was successfully retrieved using a drill dilator.Video 1

The drill dilator features a coiled sheath, a rotatable handle, and a screw-shaped, tapered tip. It is primarily used for dilation of endoscopic ultrasound-guided biliary drainage 1 2 3 . These design features allow it to pass over a wire and effectively dilate the stricture ( Fig. 2 **a, b** ). The catheter was advanced to the distal end of the stent, and a 0.025-in. guidewire (J-Wire ST, J-MIT, Japan) was inserted into the lumen ( Fig. 3 **a** ). The drill dilator was then advanced into the stent using clockwise rotation. Once engagement between the dilator and stent was confirmed, the stent was safely removed through the scope, passing beyond the stricture ( Fig. 3 **b, c** ). We confirmed the hard engagement with tension manually ( Fig. 3 **d** ).

**Fig. 2:**
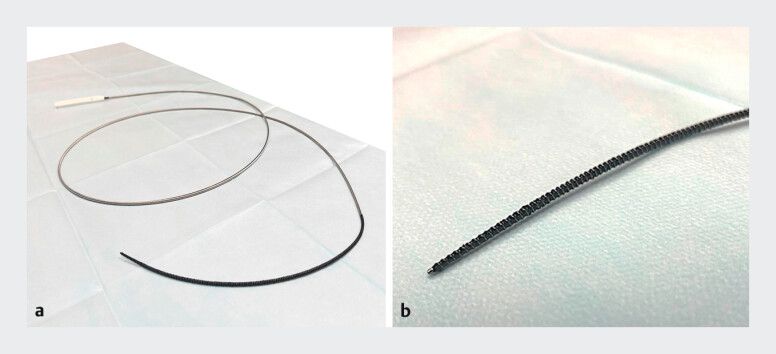
What is a drill dilator? **a** The drill dilator has a coil sheath and a rotatable handle. **b** A screw-shaped and tapered tip makes it possible to dilate the stricture through the guidewire.

**Fig. 3:**
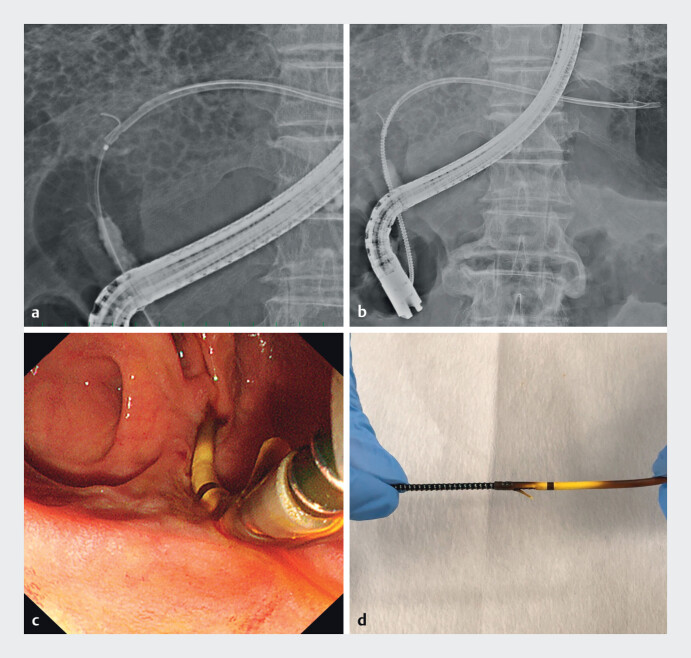
Drill dilator used for stent removal. **a** The guidewire (J-Wire ST, J-MIT, Japan) was introduced into the stent. **b** A drill dilator was inserted into the inside of the stent with clockwise rotation. **c** The stent was successfully removed over the stricture through the scope channel. **d** The drill dilator strongly involved the plastic stent.

To the best of our knowledge, this is the first report of successful retrieval using a drill dilator. This technique may serve as a viable alternative for removing intrahepatically migrated stents.

Endoscopy_UCTN_Code_CPL_1AK_2AD

## Notes

Endoscopy E-Videos https://eref.thieme.de/e-videos
*E-Videos* is an open access online section of the journal *Endoscopy* , reporting on interesting cases and new techniques in gastroenterological endoscopy. All papers include a high-quality video and are published with a Creative Commons CC-BY license. Endoscopy E-Videos qualify for HINARI discounts and waivers and eligibility is automatically checked during the submission process. We grant 100% waivers to articles whose corresponding authors are based in Group A countries and 50% waivers to those who are based in Group B countries as classified by Research4Life (see: https://www.research4life.org/access/eligibility/ ). This section has its own submission website at https://mc.manuscriptcentral.com/e-videos .
